# Myeloid differentiation primary response gene (MyD) 88 signalling is not essential for intestinal fibrosis development

**DOI:** 10.1038/s41598-017-17755-7

**Published:** 2017-12-15

**Authors:** C. Lutz, B. Weder, A. Hünerwadel, S. Fagagnini, B. Lang, N. Beerenwinkel, J. B. Rossel, G. Rogler, B. Misselwitz, M. Hausmann

**Affiliations:** 1 Department of Gastroenterology and Hepatology, University Hospital, Zurich, Switzerland; 2 Department of Biosystems Sciences and Engineering, ETH Zurich, Basel, Switzerland; 3 SIB Swiss Institute of Bioinformatics, Basel, Switzerland; 4 Institute of Social and Preventive Medicine, Lausanne University Hospital, Lausanne, Switzerland

## Abstract

Dysregulation of the immune response to microbiota is associated with inflammatory bowel disease (IBD), which can trigger intestinal fibrosis. MyD88 is a key component of microbiota signalling but its influence on intestinal fibrosis has not been clarified. Small bowel resections from donor-mice were transplanted subcutaneously into the neck of recipients C57BL/6 B6-MyD88tm1 Aki (MyD88^−/−^) and C57BL/6-Tg(UBC-green fluorescence protein (GFP))30Scha/J (GFP-Tg). Grafts were explanted up to 21 days after transplantation. Collagen layer thickness was determined using Sirius Red stained slides. In the mouse model of fibrosis collagen deposition and transforming growth factor-beta 1 (TGF-β1) expression was equal in MyD88^+/+^ and MyD88^−/−^, indicating that MyD88 was not essential for fibrogenesis. Matrix metalloproteinase (*Mmp*)9 expression was significantly decreased in grafts transplanted into MyD88^−/−^ recipients compared to MyD88^+/+^ recipients (0.2 ± 0.1 vs. 153.0 ± 23.1, respectively, p < 0.05), similarly recruitment of neutrophils was significantly reduced (16.3 ± 4.5 vs. 25.4 ± 3.1, respectively, p < 0.05). Development of intestinal fibrosis appears to be independent of MyD88 signalling indicating a minor role of bacterial wall compounds in the process which is in contrast to published concepts and theories. Development of fibrosis appears to be uncoupled from acute inflammation.

## Introduction

Innate immunity plays a pivotal role in IBD, with its two entities Crohn’s disease (CD) and ulcerative colitis (UC)^1^. In IBD, the intestinal microbiota leads to a response by the innate immune system causing inflammation. Dedicated extracellular and intracellular pattern recognition receptors, such as ten different Toll-like receptors (TLRs) and NOD-like receptors with at least 23 variants in the human genome, are responsible for sensing local microbiota^2^. TLR stimulation triggers an intracellular cascade via the cytoplasmic Toll/interleukin-1 (IL-1) receptor (TIR) signalling domain. TLR signalling depends critically on a total of four adaptor proteins — MyD88, TIR adaptor protein (TIRAP, also called MAL), (TIR)-containing adapter molecule 1 (TICAM1, also called TRIF), and TICAM2 (also called TRAM) — that directly bind to activated TLRs and recruit downstream signalling components^3^. MyD88 is a TIR domain-containing adaptor protein for the induction of inflammatory cytokines triggered by all TLRs with the exception of TLR3^3^ and is expressed in a variety of cells^4–6^. Signalling via MyD88 leads to the activation of downstream nuclear transcription factors such as nuclear factor-kB (NF-kB), cyclic AMP response element-binding protein and AP-1 ensuring the production of pro-inflammatory cytokines such as tumor necrosis factor (TNF) and IL-1, chemokines and other soluble mediators with subsequent inflammation and leukocyte accumulation^2^.

Dysregulated microbiota signalling is associated with IBD pathophysiology. Epidemiological studies of the pattern recognition receptors upstream of MyD88 clearly show an association between TLR4^7^, TLR5^8^ and TLR9^9^ polymorphisms and susceptibility to IBD. TLR1, TLR2 and TLR6 variants were found to be associated with disease phenotypes^10^. A genetic variant in the gene encoding TIRAP, necessary to recruit MyD88 to TLR2 and TLR4 has an effect on the risk of IBD^11^. Equally, for components downstream of MyD88 studies show an association between signalling dysfunction and IBD^12,13^. MyD88 can be viewed as a focal point for microbiota signalling involved in the signal transduction of almost all TLRs and causing inflammation. Studies have identified patients with mutations in *MYD88* that abolish protein production and result in a primary immunodeficiency syndrome characterized by greater susceptibility to pyogenic bacteria^14–16^. Three different autosomal recessive mutations have been described in *MYD88*-deficient patients suffering from recurrent infections with pyogenic bacteria. Life-threatening infections in subjects with defective MyD88 first occur during early infancy^14^.

Thus, one would also expect an association between MyD88 polymorphisms or its dysregulated expression and a susceptibility to IBD. A total of 163 IBD loci that meet genome-wide significance thresholds were found by analysis of data from 15 genome-wide association studies of CD and UC combined with Immunochip validation^17^. However, disease-associated MyD88 polymorphisms were not described. Further studies showed that the MyD88 polymorphism rs7744 is closely associated with the development of UC and that this polymorphism may be linked with the response to UC therapeutic approaches^18^. The function of rs7744 has not been clearly identified, although minor allele variants is thought to lead to an overexpression of the MyD88 protein^18^, considering the increased mRNA and protein levels of MyD88 in UC patients^19^.

Fibrosis is increasingly being recognised as an important cause of morbidity and mortality in IBD. While the triggering of fibrosis seems to require inflammation as a prerequisite step, the mechanisms involved in the progression of fibrogenesis may be distinct. There is evidence that intestinal fibrogenesis after being initiated is - at least to some extent - a self-perpetuating process^20^. Administration of potent anti-inflammatory agents effectively treats inflammatory flares, but at present there is no specific treatment option for patients with recurrent intestinal fibrosis^21,22^. Notable progress has been made in elucidating therapeutic targets to tackle the inflammatory component of IBD, but the incidence of strictures and of subsequent surgical interventions remains relatively unchanged^23^.

In this current study we describe the role of the MyD88-dependent innate immunity pathway in the fibrogenic process. We applied the rapid and reliable heterotopic transplantation model of intestinal fibrosis^24^ to MyD88-deficient mice. Our data suggests that the development and progression of fibrosis in the intestine is not dependent on MyD88 signalling but alludes to the presence of alternative pathways which contribute to this process. This suggests that anti-inflammatory agents – although essential to treat inflammatory flares – are not a sufficient treatment option against IBD-associated fibrosis.

## Materials and Methods

All experiments were performed in accordance with relevant guidelines and regulations.

### Animals

C57BL/6 B6-MyD88tm1 Aki (MyD88^−/−^) and C57BL/6-Tg(UBC-green fluorescence protein (GFP))30Scha/J (GFP-Tg) mice weighing 20 g were obtained from Jackson Laboratories and bred locally. The animals received standard laboratory mouse food and water *ad libitum*. They were housed under specific pathogen-free conditions in individually ventilated cages. The experimental protocol was approved by the local Animal Care Committee of the University of Zurich (registration numbers 114/2011 and ZH183/2014).

### Heterotopic intestinal transplant model

The heterotopic mouse intestinal transplant model is an adaption of the transplantation model of intestinal fibrosis in rats. Both have been previously described in detail^24,25^. In short, donor small bowel was resected and transplanted subcutaneously into the neck of recipient animals. Intestinal grafts were explanted up to 21 days after transplantation. At explantation, each graft was divided into three equal segments. One segment was fixed in 4% formalin and prepared for histopathological assessment. The other segments were snap frozen in liquid nitrogen and stored at −80 °C until RNA extraction.

### RNA isolation, cDNA synthesis and real-time PCR

For isolation of total RNA, lysis buffer from the RNeasy^®^ kit (Qiagen, Hilden, Germany) was added to snap frozen resections. Samples were shredded in M tubes (# 130-093-236, Miltenyi Biotec) in a gentle MACS^TM^ dissociator (Miltenyi Biotec). Total RNA was prepared according to the manufacturer’s protocol and stored at −80 °C. Quality of total RNA was controlled with the Agilent 2100 expert EukaryoteTotal RNA Nano Assay. Only RNA with a RNA integrity number > 6 was used for analysis. The isolated RNA was reverse-transcribed using a Reverse Transcription System (Applied Biosystems). Quantitative mRNA analysis by real-time PCR was performed using Taqman^®^ gene Expression Assays for mouse collagen type I alpha 1 (Col1a1) Mm00801666_g1, TGF-β1 Mm01178820_m1, MMP-9 Mm00442991_m1, tissue inhibitor of matrix metalloproteinase 1 (TIMP-1) Mm00441818_m1, hypoxia inducible factor 1 alpha subunit (HIF-1α) Mm01283760_m1 and GAPDH # 4352339E TaqMan® Gene Expression Assays. The relative cDNA concentration for the gene of interest was calculated using the ddCt-method.

### Immunohistochemistry (IHC)

TGF-β1 was stained with a rabbit polyclonal antibody from Santa Cruz Biotechnology Inc. (#sc-146, dilution 1:200, immunstainer Leica Bond III Stainer, pretreatment: Bond Epitop Retrival Buffer 2 for 20 min., detections kit: Bond Polymer Refine Detections Kit, all reagents from Bond Leica). The sections were examined with the Imager Z2 microscope (Zeiss) and AxioVision software (Zeiss).

Sirius Red-stained slides were analyzed by bright-field microscopy with an additional polarizing filter. Under polarized light Sirius Red-stained collagen assumes a palette of colors ranging from green to red based on the fibrotic maturation process. Collagen layer thickness was determined by an investigator blinded to the experiment. Thickness was measured in at least eight places in representative areas at 10-fold magnification. Similarly slides were stained with Elastica van Gieson and analyzed using transmission light microscopy. Collagen layer thickness was determined as previously described for Sirius Red staining.

### Statistical analysis

Statistical analysis for real time PCR and collagen layer thickness was performed using Kruskal-Wallis one way analysis of variance on ranks, all pairwise multiple comparison procedures, Dunn’s method. Comparison between genotypes was performed using the Mann-Whitney-Test for each time point. Differences were considered significant at a *p*-value of <0.05 (*) and highly significant at a *p*-value of < 0.01 (**) and *p*-value of < 0.001 (***).

### Ethical considerations

The experimental protocol for animals was approved by the local Animal Care Committee of the University of Zurich (registration numbers 114/2011 and ZH183/2014).

## Results

### Development of intestinal fibrosis is not prevented in grafts extracted from GFP-Tg donor animals and transplanted into MyD88^−/−^ recipients

To determine the relevance of MyD88 in the development of fibrosis, MyD88^−/−^ and GFP-Tg mice were used as both donors and recipients for isogeneic transplantation in our heterotopic animal model of intestinal fibrosis. The presence of endogenous GFP in GFP-Tg donor or recipient mice allowed for a genotype-specific staining. Body weight remained unchanged in both MyD88^−/−^ and GFP-Tg recipients (not shown). Grafts were explanted up to 21 days after transplantation as indicated. 26 isogeneic transplants (GFP-Tg into GFP-Tg mice), 58 isogeneic transplants (GFP-Tg into MyD88^−/−^ mice) and 47 isogeneic transplants (MyD88^−/−^ into GFP-Tg mice) were performed. Of 131 transplants histologically evaluable tissue was recovered from all but 7 grafts. To compare grafts with non-transplanted tissue 28 small bowel resections were extracted from GFP-Tg mice and six small bowel resections were extracted from MyD88^−/−^ mice.

**Figure 1:**
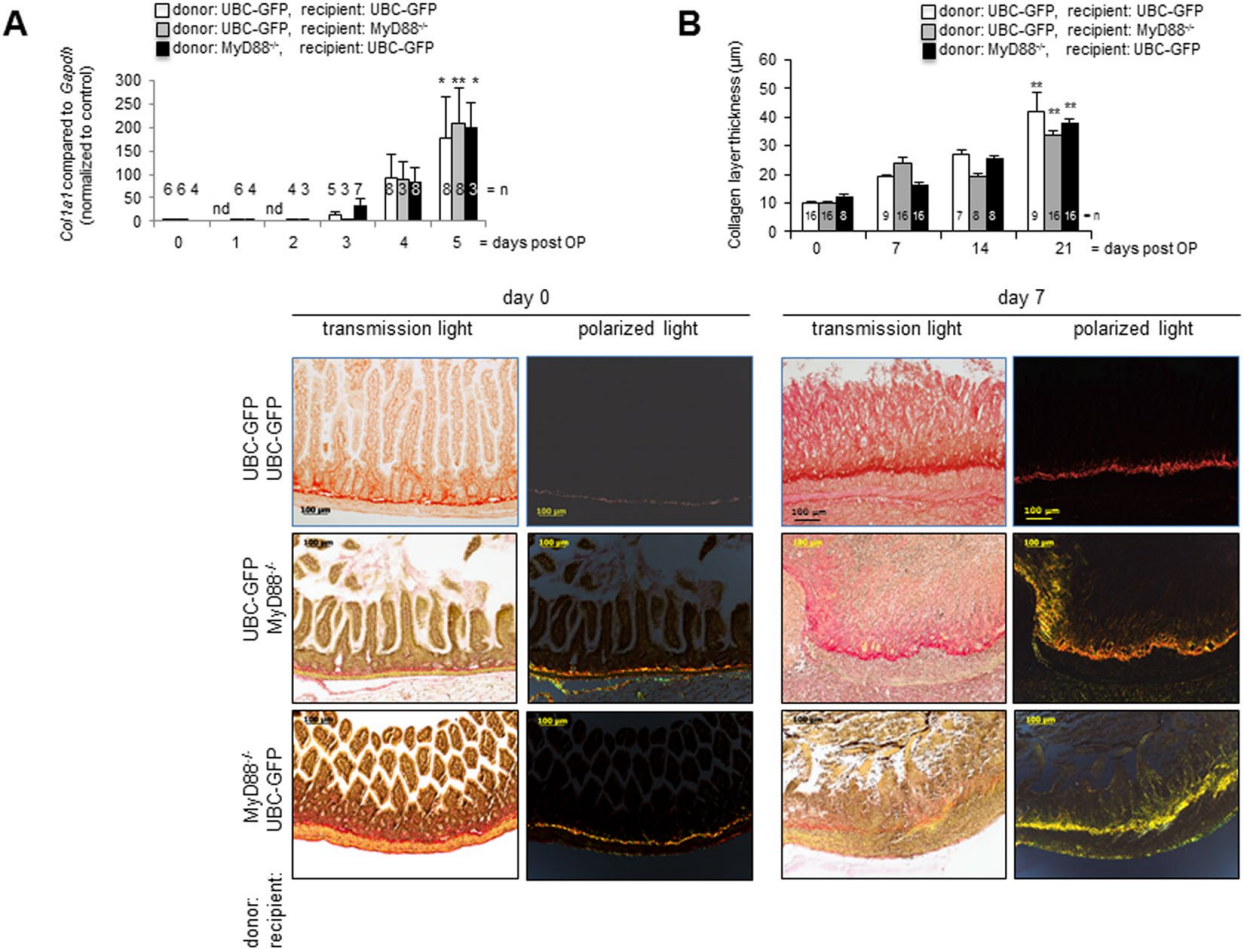
Development of intestinal fibrosis is not prevented in grafts extracted from GFP-Tg donor animals and transplanted into MyD88^−/−^ recipients. (**A**) *Col1a1* mRNA expression is significantly increased over time compared to freshly isolated intestine (p < 0.05 (*), p < 0.01(**), error bars = SEM, n = as indicated). (**B**) Sirius Red staining. Transmission light showed increased collagen layer thickness in grafts at day seven in comparison to freshly isolated intestine. Polarizing light microscopy confirmed increased collagen layer thickness at day seven. Representative figures from n = 3. (**C**) Thickness was calculated from at least eight places in representative areas at 10-fold magnification for each single graft. Collagen layer thickness increased significantly in grafts over time. Mean value and SEM is shown (p < 0.01 (**), n = 8 – 16 for each column).

Exaggerated collagen deposition is a histologic and molecular hallmark of human intestinal fibrosis, therefore *Col*1*a*1 mRNA expression was analyzed by real-time PCR (Fig. 1A). *Col*1*a1* mRNA increased in a time-dependent manner. *Col1a1* mRNA expression was significantly increased 5 days after transplantation in isogeneic transplants of GFP-Tg into GFP-Tg mice, GFP-Tg into MyD88^−/−^ mice and MyD88^−/−^ into GFP-Tg mice compared to freshly isolated small intestine (Figs. 1A, 177.3 ± 86.09 vs. 209.6 ± 73.62 vs. 1.00 ± 0.11, p < 0.01 (^**^) and 198.9 ± 52.6 vs. 0.86 ± 0.15, p < 0.05 (*) respectively, n = as indicated). The development of *Col1a1* mRNA expression curves over time is similar in grafts of both genotypes.

The development of intestinal fibrosis after heterotopic transplantation was also observed in histological cross sections. Collagen production and deposition in the intestinal transplants was determined by Sirius red staining under transmission and polarizing light microscopy (Fig. 1B). In Sirius red-stained histological cross-sections freshly isolated small intestine was characterized by an open lumen and distinctive epithelial crypts (Fig. 1B). At day 7 after transplantation the lumen of intestinal grafts was obstructed by granulation tissue and fibrotic material. Freshly isolated small intestine was characterized by mostly long-chained collagen (red stain) adjacent to the submucosa. The presence of short-chained collagen (green and yellow stain) increased continuously in the submucosa and in the luminal occlusion after transplantation. Loss of epithelial structures and luminal occlusion was observed irrespective of the genotype of donor and recipient mice. Time dependent increase in collagen was also confirmed by Elastica van Gieson (EvG) staining (Supplementary Figure 1).

The collagen layer thickness in harvested grafts was significantly increased in comparison to the collagen layer thickness in freshly isolated small intestine (Fig. 1C, p < 0.01 (**), calculated from n = number of grafts as indicated). Collagen layer thickness is comparable between grafts in both MyD88^−/−^ and GFP-Tg recipients over time.

### Myd88 is not required for an increase in *Hif1α* expression after heterotopic small bowel transplantation

**Figure 2:**
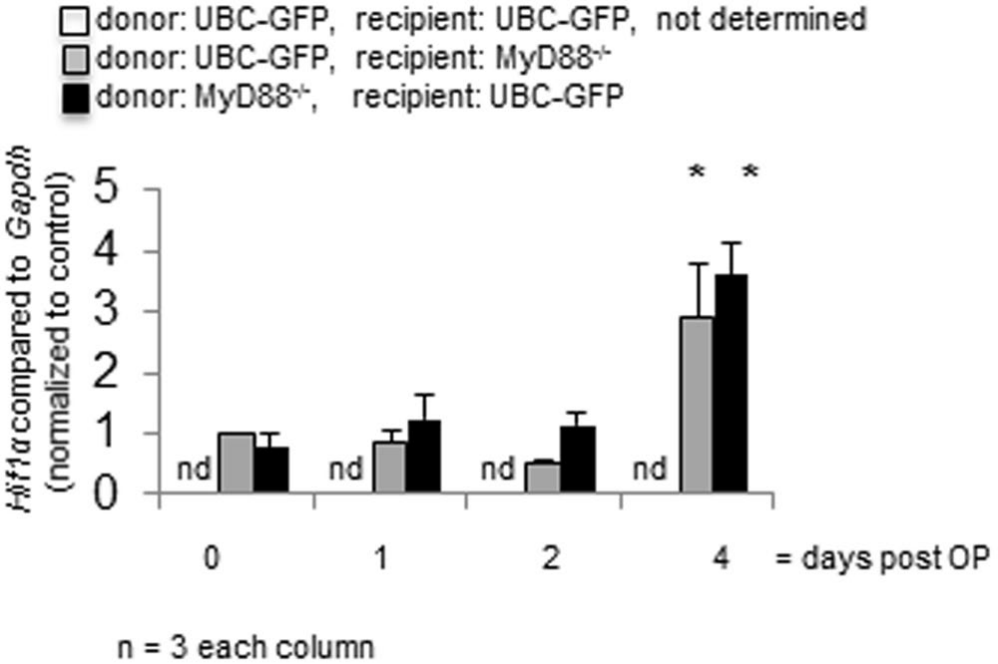
Myd88 is not required for an increase in *Hif1α* expression after heterotopic small bowel transplantation. Real time PCR confirmed significant increase of *Hif1α* expression over time (p < 0.05 (*), n = as indicated). Error bars = SEM.

MyD88 signaling regulates Hif-1α expression^26^. Therefore *Hif1α* mRNA expression was analyzed by real-time PCR to determine hypoxia-mediated signalling (Fig. 2). Surprisingly *Hif1α* mRNA was unchanged for at least 48 hours in grafts of both genotypes. This was followed by a significantly increased *Hif1α* mRNA expression after 4 days in isogeneic transplants of both GFP-Tg into MyD88^−/−^ mice as well as MyD88^−/−^ into GFP-Tg mice compared to freshly isolated small intestine (Fig. 2, 2.90 ± 0.89, vs. 1.00 ± 0.01 and 3.60 ± 0.52 vs. 0.77 ± 0.23, respectively, ^*^p < 0.05, n = 3 each column). The development of *Hif1α* mRNA expression over time is similar in grafts of both genotypes.

Previously, we could show that macroscopically visible blood vessels from the surrounding tissue extend towards the graft where they form a dense vascular network. In this study we determined the origin of the lumen-obstructing cells in isogeneic transplants (MyD88^−/−^ into GFP-Tg mice). As expected, the freshly isolated intestinal resections from MyD88^−/−^ mice are negative for GFP staining (Supplementary Figure 2A and B). An increase in infiltration of stained GFP cells ensued in a time-dependent manner. Initially, red-stained cells were visible exclusively in the submucosa and seemed to accumulate along the collagen layer. Subsequent time-points showed progressive infiltration along the crypts and into the lumen. As seen in GFP staining, the infiltrating cells frequently enter the graft not in a diffuse, but rather in a more localized fashion, with specific points of access forming along the graft wall (Supplementary Figure 2B). They then pour across the tissue and into the lumen contributing to the total occlusion of the graft. Seven days after transplantation GFP positive blood vessels appear within the GFP negative donor tissue (Supplementary Figure 2C). This result demonstrates an increased infiltration to the graft via blood vessels irrespective of MyD88-driven signalling.

### Induction *Tgfβ1* mRNA expression during fibrosis is independent from Myd88

**Figure 3:**
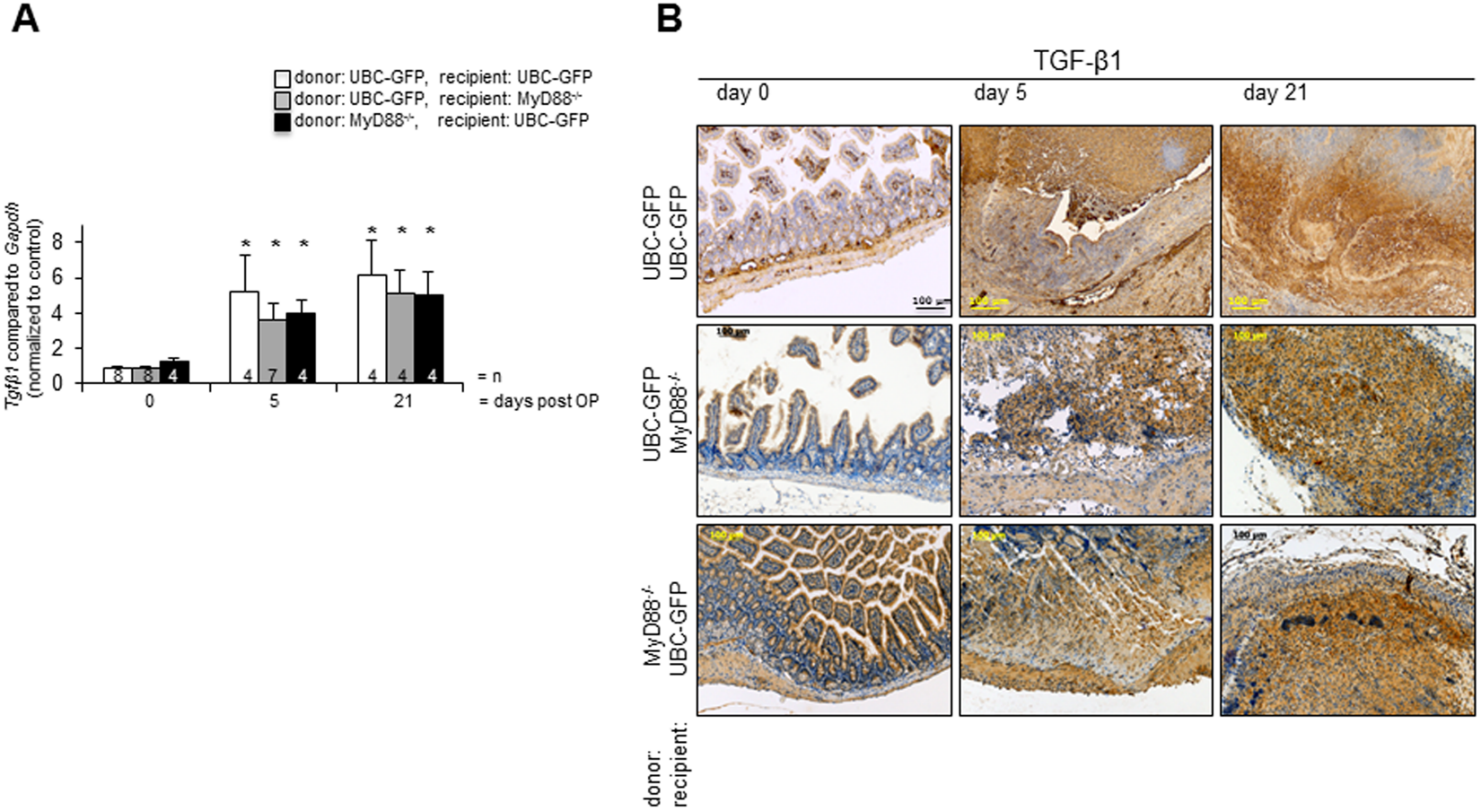
Induction of TGF-β1 during fibrosis is independent from Myd88. (**A**) Real time PCR confirmed significant increase of *Tgfβ1* expression over time (p < 0.05 (*), error bars = SEM, n = as indicated). Error bars = SEM. (**B**) IHC. In grafts a pronounced TGF-β1 staining (DAB, brown) over time was detected. Representative figures from n = 3.

To further substantiate that fibrosis evolves independently from MyD88 signalling, expression of *Tgfβ1* was analyzed. *Tgfβ1* mRNA expression was increased in a time-dependent manner (Fig. 3A). *Tgfβ1* mRNA expression was significantly increased 5 days after transplantation in isogeneic transplants of both GFP-Tg into GFP-Tg mice, in isogeneic transplants of both GFP-Tg into MyD88^−/−^ mice and MyD88^−/−^ into GFP-Tg mice compared to freshly isolated small intestine (5.20 ± 2.1, vs. 1.00 ± 0.9, 3.61 ± 0.98, vs. 1.00 ± 0.9 and 4.00 ± 0.73 vs. 1.18 ± 0.21 respectively, p < 0.05 (*), n = as indicated). Similar results were obtained 21 days after transplantation compared to freshly isolated small intestine (GFP-Tg into GFP-Tg mice: 6.20 ± 1.3, vs. 1.00 ± 0.9, GFP-Tg into MyD88^−/−^ mice: 5.17 ± 1.33, vs. 1.00 ± 0.9 and MyD88^−/−^ into GFP-Tg mice: 4.99 ± 1.42 vs. 1.18 ± 0.21 respectively, p < 0.05 (*), n = as indicated). *Tgfβ1* mRNA expression was similar between grafts of both genotypes over time. TGF-β1 was also detected by IHC along the crypt villus axis in mouse intestinal epithelial cells in freshly isolated intestine (Fig. 3B). No TGF-β1 was detected in the *lamina propria*. Intense staining for TGF was found in the luminal occlusion from day 5 to day 21 in isografts of both MyD88^−/−^ and GFP-Tg recipients.

### MyD88 deficiency reduces intestinal inflammation but not the development of intestinal fibrosis

**Figure 4:**
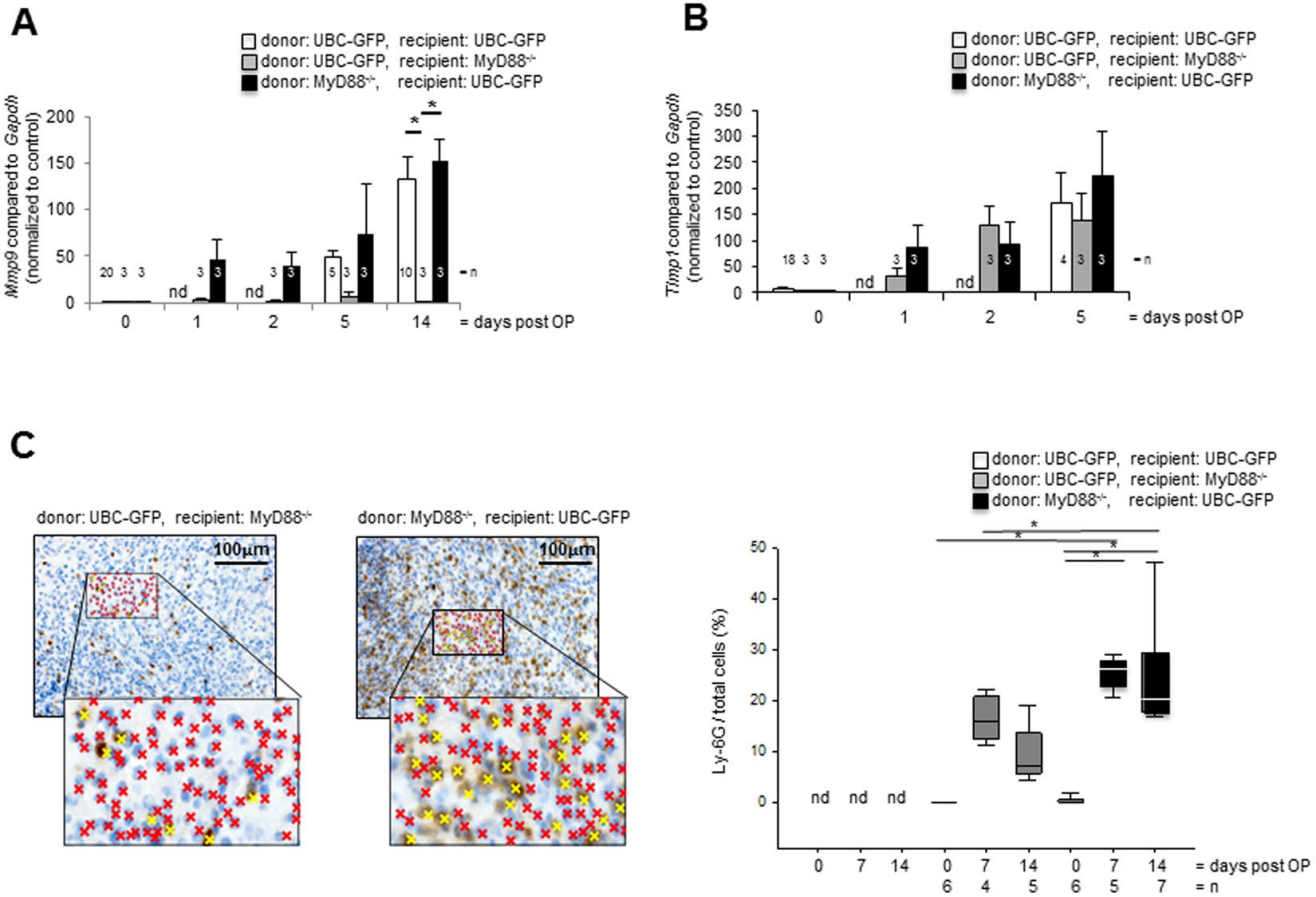
MyD88 deficiency reduces intestinal inflammation but not the development of intestinal fibrosis. (**A**) Significant increase of *Mmp9* mRNA expression in grafts isolated from GFP-Tg recipients at day 14 in comparison to freshly isolated intestine. *Mmp9* mRNA expression in grafts from MyD88^−/−^ recipients remained unchanged over time (p < 0.05 (*), error bars = SEM, n = 3 each column). (**B**) *Timp1* mRNA expression remained identical over time in both grafts from GFP-Tg and MyD88^−/−^ recipients (n = 3 each column). (**C**) IHC revealed an increase in Ly-6G^+^ neutrophils in grafts extracted from both GFP-Tg and MyD88^−/−^ donor animals compared to freshly isolated small intestine. The number of Ly-6G positive cells (yellow crosses) was significantly increased in grafts extracted from GFP-Tg (p < 0.05 (*), Kruskal-Wallis One Way Analysis of Variance on Ranks, All Pairwise Multiple Comparison Procedures, Dunn’s Method). The number of Ly-6G positive neutrophils was calculated from at least four places in representative areas at 20-fold magnification. Mean value und standard deviation is shown. n = 4 – 7 as indicated.

MyD88-signalling is an inducer of MMP-9 expression^27,28^. As MMPs are involved in mucosal fibrosis we determined the expression of tissue remodeling protease MMP-9 and the tissue inhibitor of metalloproteinases TIMP-1 in intestinal transplants by real-time PCR. *Mmp9* mRNA expression was increased in a time-dependent manner and was significantly increased in grafts from both GFP-Tg donors transplanted into GFP-Tg recipients and MyD88^−/−^ donors transplanted into GFP-Tg recipients at day 14 compared to grafts from GFP-Tg donors transplanted into MyD88^−/−^ recipients at day 14 (Fig. 4A, 133.0 ± 24.0 and 153.0 ± 23.1 vs. 0.15 ± 0.10, respectively, p < 0.05 (*), n = 3 in each column). In contrast, increase of TIMP-1 mRNA expression is similar between grafts of both genotypes over time (Fig. 4B, n = 3 each column).

As MMP-9 is produced by neutrophils in large quantities we determined if *Mmp9* mRNA expression coincided with the accumulation of Ly-6G positive neutrophils and performed the relevant immunohistochemistry. As expected, Ly-6G staining was more pronounced in grafts from MyD88^−/−^ donors transplanted into GFP-Tg recipients at day 7 and 14 compared to grafts from GFP-Tg donors transplanted into MyD88^−/−^ recipients at day 7 (Fig. 4C) and 14 (Supplementary Figure 3). A significantly increased number of Ly-6G positive neutrophils was determined in grafts from MyD88^−/−^ donors transplanted into GFP-Tg recipients at day 7 and 14 compared to freshly isolated small bowel (Fig. 4C, 25.4 ± 3.1 and 24.6 ± 10.8 vs. 0.3 ± 0.7, respectively, ^*^p < 0.05, n = as indicated). Further a significantly reduced number of neutrophils in grafts from GFP-Tg donors transplanted into MyD88^−/−^ recipients at day 7 and 14 compared to grafts from MyD88^−/−^ donors transplanted into GFP-Tg recipients confirmed the hypothesis that large quantities of MMP-9 were produced by neutrophils (Fig. 4C, 16.3 ± 4.5 vs. 25.4 ± 3.1 and 9.1 ± 5.7 vs. 24.6 ± 10.8, respectively, ^*^p < 0.05, n = as indicated). Accumulation of neutrophils was therefore significantly increased in heterotopic intestinal grafts transplanted into mice capable of MyD88 signalling compared to recipients deficient in MyD88 signalling. Therefore, MyD88 deficiency reduces intestinal inflammation, at least with respect to *Mmp9* expression and neutrophil infiltration whereas development of fibrosis is similar in grafts of both MyD88 deficient and wildtype mice.

## Discussion

In this study we investigated the role of MyD88 in the development and progression of fibrosis in an animal model of intestinal fibrosis. All hallmarks of intestinal fibrosis were similar in wildtype and MyD88^−/−^ animals. Specifically, we found increased collagen expression, which intensified over time, virtually identical in both MyD88^−/−^ and GFP-Tg grafts. Increased collagen deposition in the intestinal wall over time was detected by histological analysis and confirmed the real time PCR data. EvG and Sirius red staining demonstrated an identical network of progressively shorter-chain collagen fibrils accumulating in an IBD-mimicking fashion in grafts from both genotypes. We also found an increase in *Tgfβ1* expression over time in both grafts. Histological staining confirmed the increase in TGF-β1 in the obliterated lumen and the surrounding tissue. In addition, real-time PCR revealed a time-dependent increase of *Timp1* mRNA in both grafts.

In contrast, pronounced differences between wildtype and MyD88^−/−^ animals could also be observed. While *Mmp9* expression was increased upon development of fibrosis we did observe a significant difference between the grafts from both genotypes. MyD88-signalling is a potent inducer of MMP-9 expression^27,28^. Likewise, GFP-Tg grafts transplanted into MyD88^−/−^ recipients yielded significantly lower expression levels than the MyD88^−/−^ grafts in GFP-Tg recipients. MMP-9 is produced plentifully by neutrophils^29^, suggesting that the recipient cells infiltrating the transplant are mainly responsible for the production of this mediator. Histological staining confirms a significantly increased number of Ly-6G positive neutrophils in the obliterated lumen in grafts from MyD88^−/−^ donors transplanted into GFP-Tg recipients as compared to grafts from GFP-Tg donors transplanted into MyD88^−/−^ recipients. MMP-9 is considered as a critical component of intestinal inflammation in IBD, but less is known concerning the role of MMP-9 in penetrating or stricturing CD. Recently, we have generated an anti-MMP-9 antibody, CALY-001, and have evaluated its efficacy in fibrogenesis. We showed that inhibiting MMP-9 significantly reduces collagen deposition and intestinal tissue remodelling^30^. This anti-fibrotic effect, associated with the anti-inflammatory effect of MMP-9 inhibitor described in the DSS-colitis model by Marshall and colleagues using an alternative anti-MMP-9 antibody, AB-0046^31^, supports the development of an anti-MMP-9 antibody in patients with penetrating CD.

Several animal models for the study of intestinal fibrosis have been proposed^32^. All of them have some advantages as well as disadvantages and none of them really resembles intestinal fibrosis of CD patients. Intramural injection of peptide glycan–polysaccharide complex at laparotomy has been shown to cause intestinal fibrosis in rats with immunopathological features resembling CD^33^. In this model, bacterial cell wall polymers were shown to stimulate collagen and TGF-b1 expression in intestinal myofibroblasts^34^. In the trinitrobenzene sulfonic acid–induced model of colitis, rats develop colonic fibrosis and stricture formation, but this has not yet been well characterized^35^. Our animal model of intestinal fibrosis may have its own limitations.The observed increase in neutrophils in the heterotopic transplantation model may be affected by immune response to intestinal grafts, rejection, ischemia or hypoxia which contributes to fibrosis in the animal model but not necessarily to fibrosis in IBD patients.

Differences in the microbiome are likely to influence development of intestinal fibrosis. The intestinal microbiota that lead to a response by the innate immune system in IBD cause inflammation and may trigger fibrogenesis. However, there are conflicting reports on the importance of the MyD88-dependent signalling in the development of intestinal fibrosis. *S. typhimurium* ranges among the most prevalent agents of foodborne diseases and provokes severe inflammatory responses and thus may initiate fibrosis. Infection studies with streptomycin pretreated mice showed evidence for MyD88-dependent and MyD88-independent pathways in *S. typhimurium* colitis^36^. *S. typhimurium* was shown to efficiently colonize the cecal lumen and to cause the same level of inflammation in both MyD88^+/+^ and MyD88^−/−^^36,37^. *S. typhimurium* can efficiently induce inflammation even in the absence of MyD88 signalling. Intestinal dendritic cells are believed to sample and present not only *S. typhimurium* but all commensal bacteria to the gut-associated immune system. MyD88 signaling in mucosal dendritic cells is not required for induction of colitis by *S. typhimurium* ^38^. In contrast, using a less virulent *S. typhimurium* strain in infection studies MyD88^−/−^ mice displayed a decrease in collagen and mucosal inflammation in ceca compared to wildtype mice^37^. Submucosal inflammation and fibroblast accumulation was similar in the infected MyD88^−/−^ and wildtype mice^37^ and the authors conclude that fibrosis is not simply a secondary effect of local inflammation and tissue damage. Studies where *S. typhimurium* infection in mice was eradicated with antibiotics showed evidence that fibrogenesis is preceded by inflammation but eradication of the inflammatory stimulus represses inflammation without preventing fibrosis^20^. This suggests that intestinal fibrosis does not require persistent microbal stimulation and could be independent from the MyD88 pathways from a certain time point onwards.

There are also conflicting reports on the importance of the MyD88-dependent signalling in the development of fibrosis in other organs. Kidney fibrosis studies using various murine models have shown MyD88 to be a key component of this disease, whereby its absence coincided with reduced inflammation and attenuated collagen deposition^39,40^. MyD88-activation was linked with a shift in the immune response towards T helper type 2 differentiation and M2 polarisation of macrophages^39^. This pattern has previously been implicated in the development and progression of fibrosis^41,42^. Confirmatively, MyD88 signalling was found to be essential for the inflammation and fibrosis in a bleomycin-induced model of pulmonary disease^43^. The TLR4-triggered MyD88 pathway was found to be crucial to hepatic fibrosis by potentiating pro-fibrotic TGF-β1 signalling^44^. In contrast, in other studies using different models of fibrosis MyD88 signalling is less relevant for the development and progression of fibrosis. For instance, *Leptospira interrogans* induces fibrosis in mouse kidney in a MyD88-independent fashion^45^. Similarly, a recent study showed that MyD88, while relevant for the inflammatory process, plays only a minor role in a silica model of lung fibrosis^46^. Critically, MyD88-deficient mice presented a pronounced accumulation of pro-fibrotic cytokines and did not manifest a tempering of collagen deposition.

TLRs and NOD-like receptors are responsible for sensing local microbiota^2^. TLR are not only expressed in macrophages, dendritic cells and intestinal epithelial cells but also in primary colonic myofibroblasts^47^, one of the main producers of extracellular matrix components that are deposited during fibrosis. In mice, MyD88 is essential for the inflammatory responses mediated by almost all TLR family members^48^ as MyD88^−/−^ show no responses to the TLR2 ligands peptidoglycan and lipoprotein^49,50^, to the TLR4 ligand lipopolysaccharide (LPS)^51^, to the TLR5 ligand flagellin^52^, to the TLR7 ligand imidazoquinoline^53^ and to the TLR9 ligand CpG DNA^54,55^. On one hand MyD88 signalling is required to limit the bacterial burden and mice with a *Myd88*-deficiency have increased susceptibility to bacterial and viral infections. On the other hand MyD88-independent TLR signaling pathways have also been identified^56–58^.

Furthermore wound healing is impaired in MyD88-deficient mice. Adequate wound healing is the result of an exquisite physiological balance between multiple pro- and anti-fibrotic stimuli on extracellular matrix-producing cells^59–62^. Excessive tissue repair promotes fibrosis and impairs gastrointestinal function and is a common clinical problem in patients with IBD.

The data presented here suggest that although inflammation is reduced by MyD88 deficiency, the development of intestinal fibrosis is uncoupled from MyD88 signalling. MyD88 is not the only adaptor protein involved in Toll/IL-1 receptor family signalling. Stimulation of TLR3 and TLR4 in the absence of MyD88 still leads to the activation of NF-kB, albeit with delayed kinetics^51^. Interestingly, TLR4 polymorphisms have been associated with an increased susceptibility for IBD^63,64^. The MyD88-independent TLR4 response occurs via the adaptor TIR domain-containing adaptor inducing IFN-beta (TRIF) which then triggers the interferon regulatory factor 3 (IRF3)^57,65^ or NF-kB via RIP^66^. Furthermore, TLRs are not the only pattern recognition receptors involved in the innate immune response. Intracellular NOD/CARD-like receptors are also able to bind PAMPs^67^ and have been shown, in the case of NOD2/CARD15, to be strictly linked to CD^68^. However, even considering the presence of these alternative pathways, the acute inflammatory response will be strongly reduced without a functional MyD88, as exemplified by a strongly reduced accumulation of neutrophils in our experiments. Development of fibrosis thus appears to be uncoupled from inflammation, contradicting published concepts and theories. This suggests that anti-inflammatory agents – although essential to treat inflammatory flares – are not a sufficient treatment option against IBD associated fibrosis.

## Electronic supplementary material

Supplementary figures 1 - 3
