# Supplementary material for: Myeloid differentiation primary response gene (MyD) 88 signalling is not essential for intestinal fibrosis development
Source: Sci Rep. 2017 Dec 15;7:17678. doi: 10.1038/s41598-017-17755-7 (PMC5732165; doi:10.1038/s41598-017-17755-7)
Supplement: Supplementary file 1 — Supplementary figures 1 - 3 [file 41598_2017_17755_MOESM1_ESM.doc]

**Myeloid differentiation primary response gene (MyD) 88 signalling is not essential for intestinal fibrosis development**

Lutz C^1, *^ and Weder B^1, *^, Hünerwadel A^1^, Fagagnini S^1^, Lang B^2,3^, Beerenwinkel N^2,3^, Rossel JB^4^, Rogler G^1^, Misselwitz B^1^, Hausmann M^1, °^

^1^ Department of Gastroenterology and Hepatology, University Hospital Zurich, Switzerland

^2^ Department of Biosystems Sciences and Engineering, ETH Zurich, Basel, Switzerland

^3^ SIB Swiss Institute of Bioinformatics, Basel, Switzerland

^4^ Institute of Social and Preventive Medicine, Lausanne University Hospital, Lausanne, Switzerland

**Author Contributions**: * = CL and BW contributed equally

AH, SF: acquisition, analysis and interpretation of data.

CL, BW, BL, NB, JBR: acquisition, analysis and interpretation of data; drafting of the manuscript.

MH, BM: analysis and interpretation of data; study concept and design; drafting of the manuscript.

GR: study concept and design; critical revision of the manuscript for important intellectual content.

° Address for correspondence:

Martin Hausmann PhD

Department of Gastroenterology and Hepatology

University Hospital Zürich

University of Zurich

8091 Zurich

CH-Switzerland

Mail: [martin.hausmann@usz.ch](mailto:martin.hausmann@usz.ch)

Tel.: +41 44 255 9916


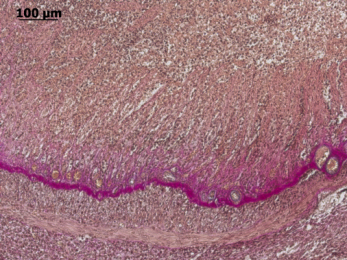

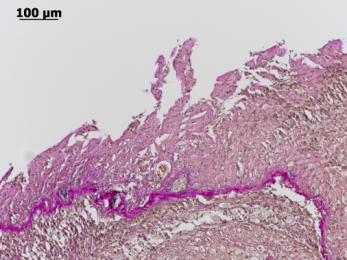

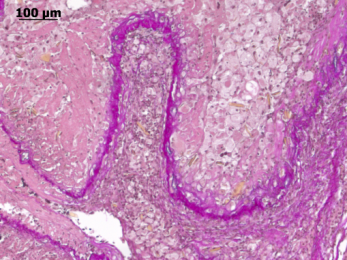

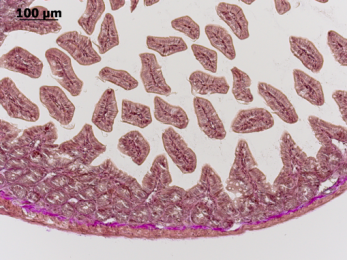


**Supplementary figure 1**

day 0 day 7 day 14 day 21

donor: UBC-GFP

recipient: MyD88^-/-^


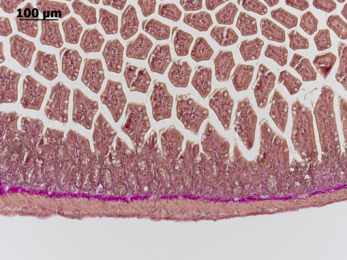

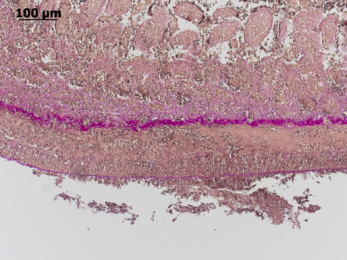

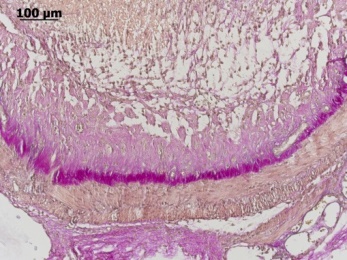

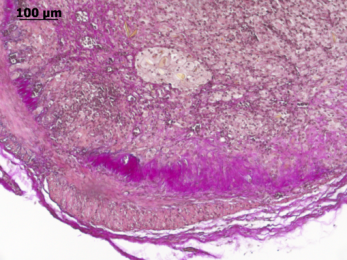


donor: MyD88^-/-^

recipient: UBC-GFP

Supplementary figure 1: **Development of intestinal fibrosis is not prevented in grafts of MyD88^-/-^ recipients.** EvG staining. Transmission light showed increased collagen layer thickness in grafts over time in both MyD88^-/-^ and GFP-Tg recipients. Representative figures from n = 3.


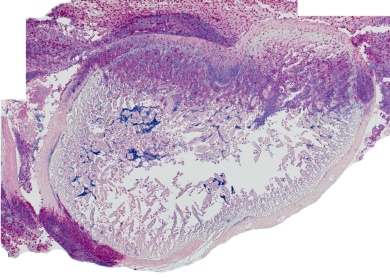


200 µm

day 0 day 2 day 4 day 7


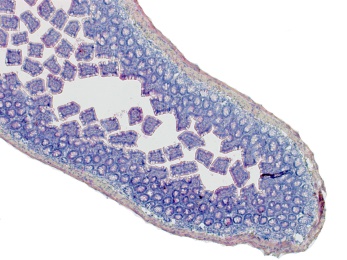

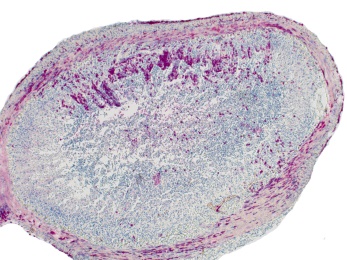

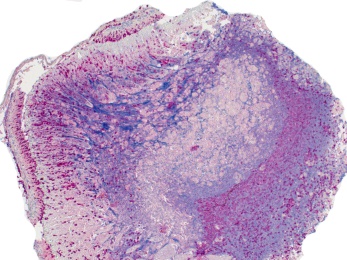


200 µm

200 µm

200 µm

**oc**

donor: MyD88^-/-^

recipient: UBC-GFP

**Supplementary figure 2 A**

Supplementary figure 2: **Host cells progressively infiltrate the intestinal graft.** IHC of lumen-obstructing cells of recipient origin. **(A)** Freshly isolated intestinal resections are negative for GFP (red stained). Increase in infiltration at day 2, 4 and 7 (as indicated) in a time-dependent manner. Infiltrating cells frequently enter with specific points of access forming across the graft wall (white arrows, original magnification 5 x). **(B)** Infiltrating cells enter the graft in a localised fashion. Dotted lines = blood vessels, oc = occluded lumen, fat = fat tissue adjacent to graft, sm = graft intestinal submocosa (original magnification 10 x). **(C)** Seven days after transplantation GFP positive blood vessels (red) including leukocytes (light brown, black arrows) appear within the GFP negative graft (original magnification 40 x).

**B**


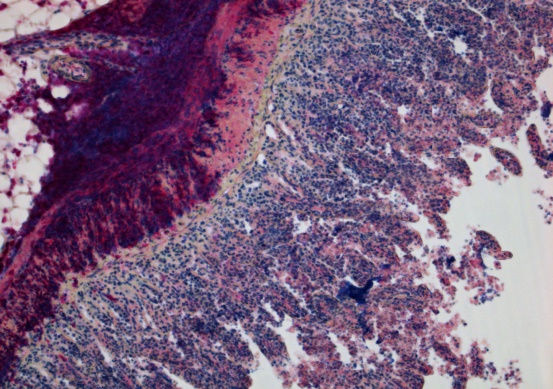


100 µm

**oc**

**sm**

**fat**

day 2

day 7


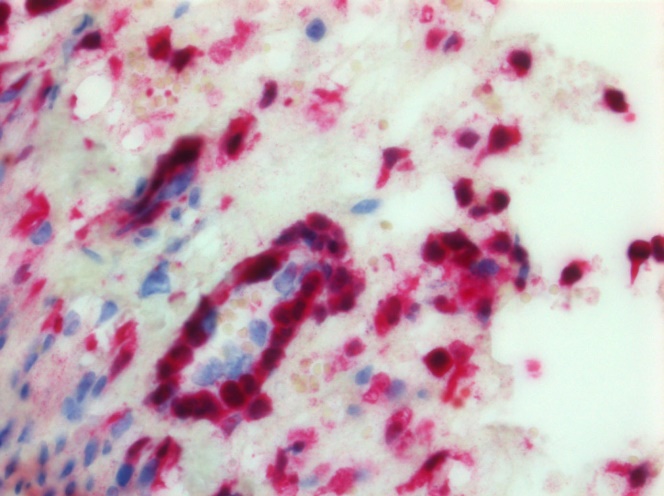


**C**

50 µm

donor: MyD88^-/-^

recipient: UBC-GFP

donor: MyD88^-/-^

recipient: UBC-GFP


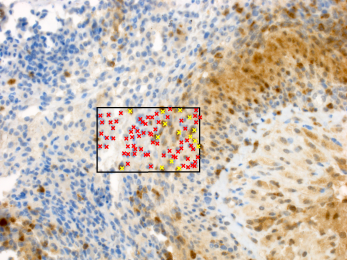

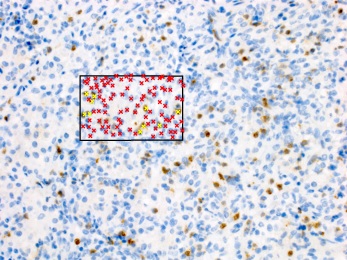

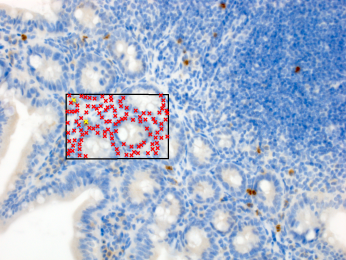

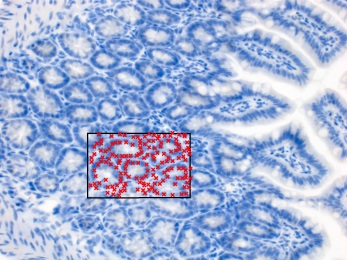

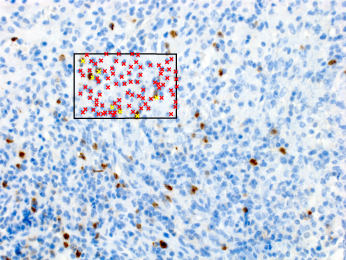

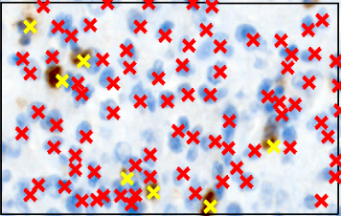

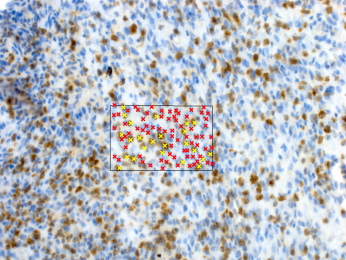

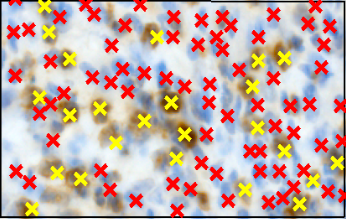

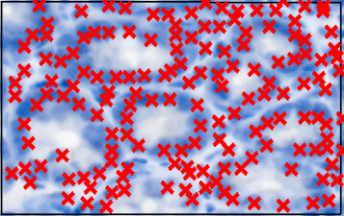

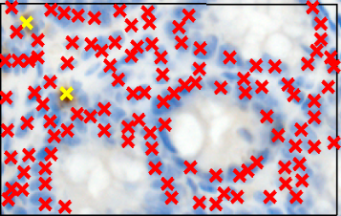

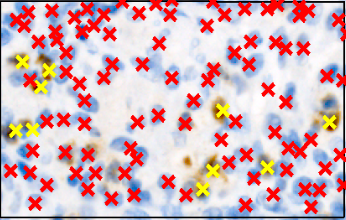

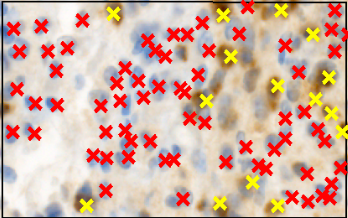


donor: UBC-GFP

recipient: MyD88^-/-^

donor: MyD88^-/-^

recipient: UBC-GFP

**Supplementary figure 3**

**100**
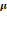
**m**

**100**
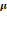
**m**

**100**
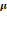
**m**

**100**
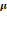
**m**

**100**
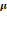
**m**

**100**
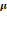
**m**

day 0 day 7 day 14

Supplementary figure 3: **Neutrophil infiltration is increased in grafts extracted from GFP-Tg recipient animals as compared to grafts from MyD88^-/-^ recipients**. IHC reveals an increase in Ly-6G^+^ neutrophils in grafts from MyD88^-/-^ donors transplanted into GFP-Tg recipients at day 7 and 14 compared to grafts from GFP-Tg donors transplanted into MyD88^-/-^ recipients at day 7 and 14.
